# Breast tumors from *CHEK2 1100delC-*mutation carriers: genomic landscape and clinical implications

**DOI:** 10.1186/bcr3015

**Published:** 2011-09-20

**Authors:** Taru A Muranen, Dario Greco, Rainer Fagerholm, Outi Kilpivaara, Kati Kämpjärvi, Kristiina Aittomäki, Carl Blomqvist, Päivi Heikkilä, Åke Borg, Heli Nevanlinna

**Affiliations:** 1 Department of Obstetrics and Gynecology, Helsinki University Central Hospital, Haartmaninkatu 8, Helsinki, FI-00029, Finland; 2 Institute for Molecular Medicine Finland, FIMM, Tukholmankatu 8, Helsinki, FI-00014, Finland; 3 Department of Clinical Genetics, Helsinki University Central Hospital, Haartmaninkatu 2B, Helsinki, FI-00029, Finland; 4 Department of Oncology, Helsinki University Central Hospital, Haartmaninkatu 4, Helsinki, FI-00014, Finland; 5 Department of Pathology, Helsinki University Central Hospital, Haartmaninkatu 3, Helsinki, FI-00014, Finland; 6 Department of Oncology, Clinical Sciences, Lund University and Skåne University Hospital, Barngatan 2B, SE 22185 Lund, Sweden; 7 CREATE Health Strategic Center for Translational Cancer Research, Lund University, BMC C13, SE 22184, Lund, Sweden; 8 Lund Strategic Research Center for Stem Cell Biology and Cell Therapy, Lund University, BMC B10, SE 22184, Lund, Sweden

## Abstract

**Introduction:**

*Checkpoint kinase 2 *(*CHEK2) *is a moderate penetrance breast cancer risk gene, whose truncating mutation *1100delC *increases the risk about twofold. We investigated gene copy-number aberrations and gene-expression profiles that are typical for breast tumors of *CHEK2 1100delC-*mutation carriers.

**Methods:**

In total, 126 breast tumor tissue specimens including 32 samples from patients carrying *CHEK2 1100delC *were studied in array-comparative genomic hybridization (aCGH) and gene-expression (GEX) experiments. After dimensionality reduction with *CGHregions *R package, *CHEK2 1100delC-*associated regions in the aCGH data were detected by the Wilcoxon rank-sum test. The linear model was fitted to GEX data with R package *limma*. Genes whose expression levels were associated with *CHEK2 1100delC *mutation were detected by the bayesian method.

**Results:**

We discovered four lost and three gained *CHEK2 1100delC-*related loci. These include losses of 1p13.3-31.3, 8p21.1-2, 8p23.1-2, and 17p12-13.1 as well as gains of 12q13.11-3, 16p13.3, and 19p13.3. Twenty-eight genes located on these regions showed differential expression between *CHEK2 1100delC *and other tumors, nominating them as candidates for *CHEK2 1100delC-*associated tumor-progression drivers. These included *CLCA1 *on 1p22 as well as *CALCOCO1, SBEM*, and *LRP1 *on 12q13. Altogether, 188 genes were differentially expressed between *CHEK2 1100delC *and other tumors. Of these, 144 had elevated and 44, reduced expression levels.

Our results suggest the WNT pathway as a driver of tumorigenesis in breast tumors of *CHEK2 1100delC-*mutation carriers and a role for the olfactory receptor protein family in cancer progression. Differences in the expression of the 188 *CHEK2 1100delC-*associated genes divided breast tumor samples from three independent datasets into two groups that differed in their relapse-free survival time.

**Conclusions:**

We have shown that copy-number aberrations of certain genomic regions are associated with *CHEK2 *mutation *1100delC*. On these regions, we identified potential drivers of *CHEK2 1100delC-*associated tumorigenesis, whose role in cancer progression is worth investigating. Furthermore, poorer survival related to the *CHEK2 1100delC *gene-expression signature highlights pathways that are likely to have a role in the development of metastatic disease in carriers of the *CHEK2 1100delC *mutation.

## Introduction

Large-scale gene-expression (GEX) profiling by DNA microarrays has become a routine method in breast cancer research. It has been widely used in tumor-subtype classification, and gene signatures predicting clinical outcome have been defined in a number of studies [1-8].

The tumor genome is characterized by multiple translocations as well as gains and losses of chromosomal regions. Overall chromosomal instability is a result of arbitrary processes that take place during tumor progression, like breakage-fusion-bridge cycles [9], spontaneous breaks [10], and aberrant segregation of chromosomes in mitosis [11]. The cells that by chance gain advantageous changes survive and form a new more-malignant subpopulation of cancer cells.

Array-comparative genomic hybridization (aCGH) enables fine mapping of the locations of chromosomal breakpoints and detection of copy number on each location [12,13]. Combined aCGH and GEX profiling represents a valuable tool. aCGH can detect the events that have been selectively advantageous during the tumor progression as consistently gained or lost regions across a set of samples. GEX can further highlight the driver genes within these regions, revealing the ones whose expression levels have changed [14].

Understanding cellular processes that drive tumorigenesis is essential to select the optimal treatment for cancer [15]. Breast cancer can be divided into several subtypes that are characterized by differences in histopathologic features [16] as well as in genomic copy number [17,18] and gene-expression profiles [1,5,19,20]. These differences ultimately derive from different biologic pathways that drive the tumor progression. For example, tumors from *BRCA1- *and *BRCA2-*mutation carriers exhibit distinct copy-number and gene-expression profiles and cluster into separate breast cancer subtypes [2,18,21], suggesting that events taking place during tumorigenesis are different and are influenced by the underlying inherited vulnerability of the key players, known as breast cancer tumor-suppressor genes. This raises a possibility also that moderate-penetrance mutations, such as *CHEK2 1100delC*, could be associated with certain genomic changes and gene-expression profiles.

*CHEK2 *(checkpoint kinase 2) is an intermediate-level breast cancer risk gene, whose truncating mutation *1100delC *doubles the risk of unselected women [22], but gives rise to much higher risk for women who have a family history of breast cancer [23]. Frameshift mutation *1100delC *causes premature translation stop at codon 381 in the middle of the kinase domain of the protein [24]. Truncated protein is highly unstable [25,26] as well as the mutated mRNA, which is rapidly degraded through nonsense-mediated mRNA decay [27].

The well-known role of CHEK2 is to regulate cellular responses to DNA double-strand breaks (DSBs). ATM (ataxia telangiectasia mutated) phosphorylates CHEK2 in response to DNA damage. This leads to CHEK2 homodimerization, resulting in an active kinase whose targets include cell-cycle regulators CDC25A, CDC25C, PLK and E2F1, BRCA1 involved in homologous recombination, as well as the master coordinator of apoptosis, TP53 [28]. Recently CHEK2 was reported to be involved also in regulation of the proper assembly of the mitotic spindle [29]. Even partial loss of CHEK2 activity was sufficient to cause chromosomal instability.

Loss of 22q, where the *CHEK2 *locus resides, is a common event in breast cancer [30,31]. Tumors from patients carrying *CHEK2 *mutation *1100delC *show reduced CHEK2 protein activity that can partly be explained by the loss of the wild-type allele in tumor cells [25,32]. However, loss of heterozygocity (LOH) does not comprehensively explain *CHEK2 1100delC-*related tumorigenesis, and likely also other molecular mechanisms exist, through which *CHEK2 *deficiency contributes to malignant development [26,33]. *CHEK2 1100delC *mutation is associated with estrogen receptor (ER) positive and higher grade tumors, as well as with bilateral disease [34,35]. Breast cancer patients with *CHEK2 1100delC *have been found to have a poorer disease-free and overall survival [34,36], and this may partly be due to a disadvantageous response to treatment that causes DNA damage [37,38].

Here we report the first study investigating *CHEK2 1100delC-*related tumorigenesis by comparing gene-expression profiles and genomic changes in breast tumors of *CHEK2 1100delC-*mutation carriers to those of patients negative for any known germline mutations. As CHEK2 deficiency is not restricted to tumors of *1100delC-*mutation carriers [25], similar copy number aberrations and gene-expression changes could also be present in the tumors of noncarriers, but enriched in the mutation carriers. The genomic aberrations as well as activated or silenced genes and pathways characteristic for *CHEK2 1100delC *mutation-carrier tumors are good candidates for *CHEK2 1100delC-*related tumor-progression drivers to be investigated further. Finally, we investigated the clinical significance of the discovered *CHEK2 1100delC *gene-expression signature.

## Materials and methods

### Patients and tumor tissue samples

In total, 126 tumor tissue samples from 121 breast cancer patients negative for *BRCA1, BRCA2*, or *TP53 *germline mutations were obtained from Helsinki University Central Hospital, Department of Pathology. Thirty of the patients carried germline mutation *CHEK2 1100delC*. Tumor characteristics are described in detail in additional files (Additional file 1, Additional file 2).

The study was carried out with the permission from the Helsinki University Central Hospital Ethics Committee (Dnro207/E9/07) and with written informed consents from the patients. Both processed and raw data of aCGH and GEX experiments are available in Gene Expression Omnibus database [GEO: GSE24707] [39].

### Gene-expression microarrays

Labeled tumor cDNA samples and reference samples (Universal Human RNA; Stratagene, La Jolla, CA, U.S.A.) were hybridized to custom-made cDNA microarrays [GEO: GPL5345] produced at SCIBLU Genomics Centre, Lund University, Sweden, as described earlier [40]. Two samples were hybridized twice, and one sample, 3 times for quality control. Nucleic acid extraction, data acquisition, and preprocessing are described in detail in additional files (Additional file 2, Additional file 3).

### Comparative genomic hybridization microarrays

The tiling-resolution genomic BAC (bacterial artificial chromosome) arrays [GEO: GPL4723] were described in detail previously [40], along with the slide treatment and hybridization protocol. Further details on nucleic acid extraction, hybridizations, data acquisition, preprocessing, and quality control are described in detail in additional files (Additional file 2, Additional file 3, Additional file 4).

### aCGH data analysis

Data analysis was carried out in R software environment for statistical computing version 2.10. Soft calls (that is, loss, normal, and gain with probabilities) were calculated for each clone of the segmented data with the package *CGHcall *[41]. Sequences of clones with constant calls across all samples or across the *CHEK2 1100delC-*mutation carrier samples were defined by using R package *CGHregions *[42], allowing maximal information loss of 2.5%. Regions defined across all samples were tested for significant differences in calls between *CHEK2 1100delC-*mutation carrier and other tumors with the Wilcoxon rank-sum test, and those with nominal *P *values less than 0.01 were considered to be of interest. To assure that the results were not confounded by the estrogen receptor (ER) status, we performed similar analysis comparing ER-positive and ER-negative tumors to discover ER-associated regions. Regions defined only on basis of the *CHEK2 1100delC-*mutation carrier tumor samples by *CGHRegions *analysis were used primarily to explore the copy number of the genomic region covering the *CHEK2 *locus.

### Gene-expression data analysis

Gene-expression data analysis was carried out in R version 2.10 package *limma *[43-45]. For comparison of *CHEK2 1100delC *carrier and other tumors using multivariate analysis, a linear model with six covariates was fitted in the data. Specifically, as ER status is one of the most important tumor characteristics, which has a great impact also on the tumor gene-expression profile, we included it as a covariate in the multivariate linear model estimating the associations of the covariates on gene-expression levels in our dataset. The algorithm estimated which proportion of gene-expression changes was associated with *CHEK2-*mutation carrier status (independent of ER status), and simultaneously, which proportion of gene expression was associated with ER status (independent of *CHEK2 *mutation status) and similarly for all covariates. In addition to the tumor ER status and *CHEK2 1100delC*, the six covariates included a family history of breast cancer, tumor histopathologic types ductal or lobular, as well as *NQO1 rs1800566 *genotype, for its rare allele has been associated with poor breast cancer survival [46], and the gene is likely to have a role in tumor progression. Frequencies of other histopathologic types were too low to have any detectable effect. For five samples, data for all covariates was not available, and these samples were omitted from the multivariate analysis. Statistical significance of differentially expressed genes was assessed with the empiric bayesian method. Genes with nominal *P *values less than 0.05 were considered to be differentially expressed. This resulted in a set of 862 genes that were used for functional enrichment analysis. The 188 genes used in the survival signature were selected by using a stricter *P*-value threshold, 0.01.

### Functional annotation of differentially expressed genes

Functional annotation and enrichment analyses were carried out with the DAVID microarray functional annotation tool [47] and with the AmiGO [48] version 1.7 from the Gene Ontology [49,50] database during the period of July to October 2010, as well as from published literature. We also compared the *CHEK2 1100delC-*related list of 188 genes with previously published breast cancer gene-expression signatures designed for either subtype classification or survival prediction to detect possible similarities [1-8].

### Survival analysis of public datasets

The survival effect of 188 *CHEK2 1100delC-*related genes was studied in a larger Helsinki gene-expression dataset of 183 unselected and familial breast tumor samples [GEO: GSE24450] as well as in three public datasets with the approach previously suggested [51]. The Helsinki dataset consisted of 151 breast cancer patients collected in three unselected cohorts previously described [35,46,52] and 32 additional familial breast cancer cases. The three public datasets consisted of two Swedish cohorts, one from Uppsala [GEO: GSE3494; GEO: GSE4922] [53,54] and another from Stockholm [GEO: GSE1456] [55]. Differences in sample selection and survival-analysis end points are summarized in additional files (Additional file 5). Of the 188 *CHEK2 1100delC-*related genes, 131 (69.7%) were present on the array used for the Helsinki dataset, and 108 (57.4%) on the array used for all Swedish datasets. The gene-expression matrices for the available genes were used as input of k-means clustering implemented in R v.2.11, with k = 2 and using 100,000 iterations to split the samples of each dataset into two groups based on expression of the signature genes. Stabilization of the k-means results was secured by setting a random-number-generator schema for the initial k-means iteration. Differences in survival between the two groups of each dataset were evaluated by the log-rank test, and the Kaplan-Meier survival curves were generated for visualization.

## Results

### Areas with differing copy number

**Figure 1:**
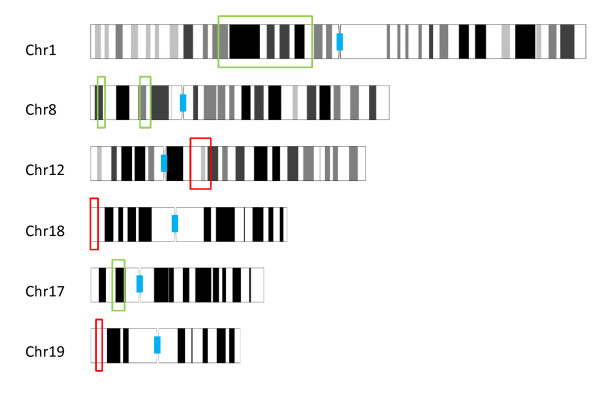
**Regions of differing copy number between *CHEK2 1100delC-*mutation carriers and controls**. Chromosomal (Chr) locations of lost regions typical of *CHEK2 1100delC-*mutation carriers are marked with green boxes, and gained regions, with red boxes.

In the following sections, we use "copy number" to refer to the following three states: loss, normal, or gain. Seven chromosomal locations were found to differ in copy number between the *CHEK2 1100delC*-mutation carrier and other tumors, as defined by *P *values less than 0.01 when tested with the Wilcoxon rank-sum test. *CHEK2 1100delC-*associated regions included wide loss of 1p13.3-31.3, losses of 8p21.1-2, 8p23.1-2, and 17p12-13.1, as well as gains of 12q13.11-3, 16p13.3, and 19p13.3 (Figure 1, Table 1). The association with the *CHEK2 *mutation was not confounded by the ER status (data not shown).

**Table 1:** Chromosomal regions with differing copy number between *CHEK2 1100delC-*mutation carriers and others

Chromosome	Start	End	Clones	Sum of clones	Length	Cytoband	*P *value
1	63762320	75867880	109	410	46865220	1p13.3-31.3	0.0031
1	76025260	87854764	110				0.0004
1	87964559	98030482	81				0.0001
1	98201778	110627540	110				0.0004
8	3329536	5083904	26	46	4279506	8p21.1-2	0.0062
8	5292190	6859255	15				0.0027
8	7041464	7609042	5				0.0066
8	24208215	29092984	35	41	5366218	8p23.1-2	0.0035
8	29293167	29574433	6				0.0064
12	46370995	47105108	10	103	9563151	12q13.11-3	0.0018
12	47191262	49654440	21				0.0022
12	49858115	51459707	21				0.0065
12	51492453	52261807	9				0.0025
12	52382320	54215964	23				0.0009
12	54367089	55934146	19				0.0044
16	11139	3142783	23	23	3131644	16p13.3	0.0091
17	9578676	15297799	54	54	5719123	17p12-13.1	0.0025
19	2122188	4798764	17	17	2676576	19p13.3	0.0045

Areas on chromosomes 1, 8, and 12 consist of multiple non-overlapping regions with differing copy numbers, whereas areas on chromosomes 16, 17, and 19 consist of single regions. Each row represents one region with constant calls across all samples. Sum of clones and length columns give the number of clones on all regions of each area and the total length of the area, respectively.

### The genomic region of *CHEK2 *locus in mutation-carrier tumors

The genomic region, which covered the *CHEK2 *locus and where all *CHEK2 1100delC *samples had consistent calls, as defined by CGH regions analysis, spanned three successive BAC clones, almost 2 kb, and five genes. This region was lost in six, gained in four, and of normal copy number in 12 of the *CHEK2 1100delC *samples.

### Gene-expression analysis

To estimate the independent effect of *CHEK2 1100delC*, we fitted a multivariate linear model on the gene-expression data and included the tumor ER status and histopathologic type as well as patient's family history of breast cancer and rs1800566 genotype as covariates. Comparison of gene-expression data from *CHEK2 1100delC-*mutation carrier and other tumors resulted in 188 (Additional file 6) and 862 differentially expressed genes with nominal *P*-value thresholds 0.01 and 0.05, respectively. Fold changes ranged from half to almost fourfold (Additional file 7). In the gene set resulting from the more stringent *P*-value cut-off, 144 genes had increased, and 44, reduced expression, and in the total of 862 genes, the respective numbers were 522 and 340.

### Enriched functional groups among differentially expressed genes

The most significantly enriched functional group among the set of 862 differentially expressed genes was olfaction: altogether, 35 olfactory receptors (OR), 34 of them having elevated expression in *CHEK2 1100delC *tumors, were found (Additional file 7, Additional file 8). The 35 OR genes were located on 10 different chromosomes. Of these, 16 resided on chromosome 11, clustering in two blocks: 10 genes on 11p15.4 and five genes on a region flanking the centromere (11p11.2-11q12.1). Furthermore, we identified two chromosomal regions where more than two differentially expressed OR were located together with other differentially expressed genes, 6p21.33-6p22.1 and 12q13.11-12q13.3.

Other interesting enriched biologic entities among the set of genes with elevated expression (Additional file 8) were the interrelated WNT signaling pathway, cell adhesion, and calcium binding (Additional file 9). Also transcription regulation-related genes and members of zinc-finger, Ras-GEF, and EGF families were enriched.

In the set of genes with lower expression in *CHEK2 1100delC *tumors, the enriched functions (Additional file 10) included DNA modification, transcription, mRNA processing, ribosome, and translation. Additionally, we retrieved several genes related to mitochondria and cellular respiration, as well as to the cytoskeleton. Some genes involved in the recognition of DNA damage were downregulated as well.

### Differentially expressed genes on regions with differing copy numbers

Significantly differentially expressed genes were found on four of the seven *CHEK2 1100delC-*associated regions: those on chromosomes 1, 8, 12, and 16. The overall level of gene expression in the *CHEK2 1100delC-*associated regions was not consistent with the corresponding copy number, which was as expected, because only partial overlap was noted between the sample sets in aCGH and GEX experiments. However, we identified a handful of genes as candidates driving *CHEK2 1100delC-*related tumorigenesis. These included β-catenin-mediated transcription-related *CALCOCO1 *[56], breast cancer poor-prognosis marker *SBEM *[57,58], and *SMARCC2*, a member of SWI/SNF chromatin-remodeling complex interacting with BRCA1 [59]. All were located on 12q13 and showed elevated expression levels in *CHEK2 1100delC *tumors.

*CLCA1 *on 1p22.3 was among the top-ranking genes of differential expression, but its expression level was inconsistent with the *CHEK2 1100delC *typical copy number, for the region was frequently lost (Table 2).

**Table 2:** Differentially expressed genes on regions with differential copy number

Gene symbol	GEX: fold change	GEX: *P *value	Region	aCGH: gain/loss
*CLCA1*	1.8178	0.000007	chr1	Loss
*DPH5*	0.8254	0.0049	chr1	Loss
*C1orf62*	1.3277	0.0057	chr1	Loss
*ATXN7L2*	1.2804	0.007	chr1	Loss
*DR1*	0.8474	0.0108	chr1	Loss
*TMED5*	0.8728	0.0145	chr1	Loss
*ALG14*	0.8640	0.0389	chr1	Loss
*LRRC8D*	0.8854	0.0433	chr1	Loss
*DEFA5*	1.1076	0.0113	chr8	Loss
*DUSP4*	1.2724	0.0426	chr8	Loss
*OR6C3*	1.2558	0.0016	chr12	Gain
*CALCOCO1*	1.2579	0.0016	chr12	Gain
*SBEM*	3.0936	0.0217	chr12	Gain
*OR6C2*	1.1820	0.0485	chr12	Gain
*LRP1*	0.8171	0.0011	chr12	Gain
*OR10AD1*	1.2648	0.0061	chr12	Gain
*CSAD*	1.2758	0.0067	chr12	Gain
*C12orf10*	0.8867	0.0122	chr12	Gain
*MYO1A*	0.7468	0.0131	chr12	Gain
*LALBA*	1.2369	0.0187	chr12	Gain
*ITGA7*	1.2531	0.0201	chr12	Gain
*OR9K2*	1.4794	0.0231	chr12	Gain
*KRT5*	1.7237	0.0382	chr12	Gain
*FAM112B*	0.7840	0.0426	chr12	Gain
*SMARCC2*	1.1788	0.047	chr12	Gain
*KIAA1924*	0.8549	0.0156	chr16	Gain
*NTHL1*	0.8410	0.0233	chr16	Gain
*KIAA1171*	1.1890	0.0348	chr16	Gain

Five of eight differentially expressed genes on 1p13.3-31.3 show reduced expression levels. On 12q13.11-3, 11 of 15 genes show elevated expression. aCGH, array comparative genomic hybridization; GEX, gene expression.

### Survival effect of 188 differentially expressed genes

**Figure 2:**
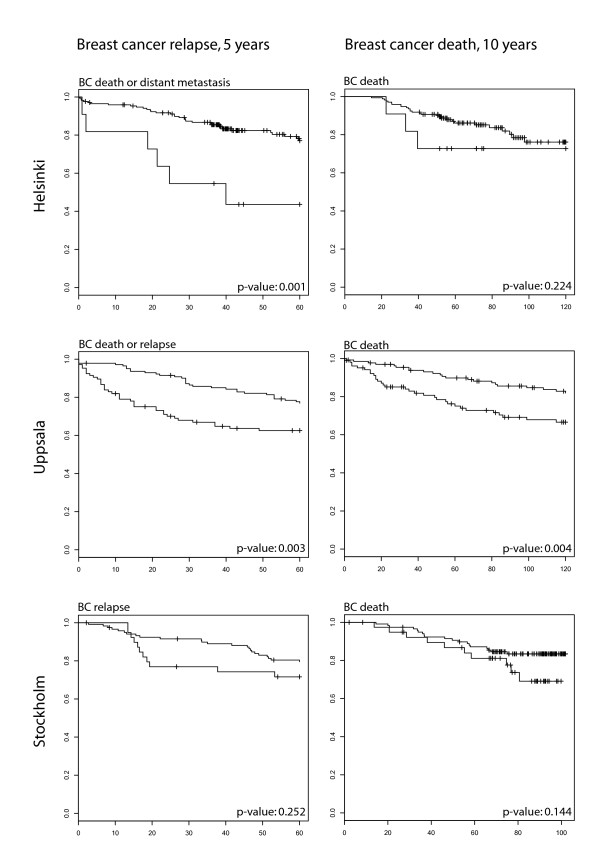
**Breast cancer (BC)-related survival differences in three sample sets divided on the basis of the *CHEK2 1100delC *signature**. Expression levels of the *CHEK2 1100delC-*related genes divide breast tumors into groups of better and poorer prognosis. Significant differences in overall or disease-free survival were detected in Helsinki and Uppsala cohorts, whereas in the Stockholm cohort, the differences were more subtle. The difference between the curves was measured with the log-rank test.

We further tested whether the 188 *CHEK2 1100delC-*related genes (Additional file 7) might be associated with breast cancer prognosis. Based on their expression levels in the Helsinki and Uppsala gene-expression data sets, it was possible to divide the tumors into groups with different survival rates of the patients. Also in the third one, the smaller Stockholm dataset, there was evidence for survival difference, but it did not reach statistical significance. (Figure 2). Altogether, the *CHEK2 1100delC *gene-expression signature was associated with more rapid relapse of the disease in all three sample sets. In the largest data set (Uppsala), the *CHEK2 1100delC *signature also significantly also predicted 10-year breast cancer-specific survival.

Little similarity was seen between the gene list of 188 *CHEK2 1100delC-*associated genes and eight gene lists from previously published gene-expression signatures predicting survival or breast cancer subtype (Table 3). Not more than about 1% of genes from any previously published gene list were shared by the *CHEK2 1100delC-*related list.

**Table 3:** Overlap between the 188 *CHEK2 1100delC-*associated genes and eight previously published gene-expression signatures

Gene-expression signature	Genes	Overlap	Proportion
SSP2003 [2]	500	5	0.010
SSP2006 [1]	306	2	0.007
PAM50 [5]	50	1	0.020
70 gene poor-prognosis signature [3]	70	0	0.000
76 gene metastasis signature [4]	76	0	0.000
Gene-expression grade index [6]	128	1	0.008
Recurrence score [7]	21	0	0.000
Recurrence predictor in tamoxifen-treated patients [8]	181	2	0.011
GO: proliferation [49,50]	1677	13	0.008

The first column contains names and references for the gene-expression signatures as well as the reference for proliferation-associated genes in the Gene Ontology database (GO). The second to fourth columns indicate the number of genes included in the signature, the number overlapping with each signature, and the 188 genes and proportion of genes of each signature among the 188 genes, respectively.

## Discussion

We investigated the genomic copy number and gene-expression profiles of breast tumors of *CHEK2 1100delC-*mutation carriers. We aimed to identify patterns enriched in the *CHEK2 1100delC *group compared with the control group, to understand tumorigenesis, to which the *CHEK2 *germline deficiency predisposes. We discovered two larger and five more narrow genomic regions whose copy-number aberrations were typical of *CHEK2 1100delC *tumors. We also identified 862 genes whose expression levels differed between the two groups of tumors, suggesting differential activity of especially WNT and olfactory pathways in these groups. Furthermore, we observed that the *CHEK2 1100delC *gene-expression signature is related to increased risk of breast cancer relapse.

Among the genes with lower expression levels in *CHEK2 1100delC *tumors, we observed several genes associated with centrosomes and the cytoskeleton or with DNA-damage signaling and apoptosis, as expected, owing to the role of CHEK2 in response to DNA damage and in centrosome assembly. The apoptosis-related genes included pro-apoptotic *BAX *and *BAD *and DNA damage sensor *PARP1*.

BCL-2 family proteins, including BAD and BAX, are involved in the regulation of the normal breast tissue development and involution. Their expression is often disturbed in malignant tissue, and reduced expression of BAD and BAX is associated with poor prognosis in breast cancer. All BCL-2 proteins regulate the mitochondrial apoptotic pathway. BAX induces apoptosis by increasing the permeability of the mitochondrial outer membrane to release cytochrome *c*. BAD is an upstream regulator of several pro- and antiapoptotic BCL-2 family proteins, BAX among them [60]. *BAX *is a transcriptional target of TP53 [61] and therefore also a downstream target of CHEK2. Its downregulation could be seen as a consequence of reduced CHEK2 activity, but at the same time, as a survival mechanism of the tumor cells.

aCGH analysis highlighted seven genomic locations, whose copy-number aberrations were enriched in tumors of *CHEK2 1100delC-*mutation carriers. These included losses of the proximal part of 1p, two regions on 8p and a narrow region in the middle of 17p as well as gains of the proximal region on 12q and short distal regions on 16p and 19p. Loss of 8p is a common aberration in breast tumors, and several loci on 8p have been suggested to harbor tumor-suppressor genes [62]. Our data support these reports. The entire chromosome arm 8p was frequently lost in both *CHEK2 1100delC *and other tumors, but losses of certain sections of the arm were more frequent in *CHEK2 1100delC *tumors. Similarly, a region distal to 1p31 has been suggested to harbor tumor-suppressor genes, because it is commonly lost in breast and other solid tumors, and the loss is an early event in breast carcinogenesis [63]. Interestingly, this region covers only a small proportion of the *CHEK2 1100delC *associated more proximal to 1p13.3-31.3. Expression of a handful of genes located on 1p13.3-31.3 differed between *CHEK2 1100delC-*carrier tumors, the most significant of them being the calcium-activated chloride channel, *CLCA1*. *CLCA1 *and its three paralogs *CLCA2*, -*3*, and -*4*, all reside on 1p22.3. *CLCA2 *that shares 63% sequence similarity with *CLCA1 *[64] has been suggested to be a breast cancer tumor-suppressor gene [65]. The *CLCA1 *locus was lost in one third of *CHEK2 1100delC *tumors but only in 3.5% of other tumors. Conversely, *CLCA1 *gene expression was significantly higher in *CHEK2 1100delC *tumors. From our data, we cannot state whether the loss of the *CLCA1 *locus and elevated expression were simultaneous events. They could also be alternative events both affecting CLCA1 normal function.

12q13 is a locus of a *WNT *gene cluster, hosting genes *WNT10B *and *WNT1*, as well as WNT pathway modifiers *CALCOCO1 *and *LRP1 *[66]. It is also a locus for a cluster of olfactory receptor genes. The results from GEX and aCGH analyses converge to highlight the importance of these two pathways. Expression of altogether 15 genes related to the WNT pathway was elevated. These included such central players as *WNT2 *growth factor and *c-MYC *cellular oncogene. Expression differences of olfactory receptor genes were even more pronounced: up to 34 OR genes had higher expression in *CHEK2 1100delC-*mutation carriers than in the control samples.

In adult human breast tissue, WNT2 expression is restricted to stromal cells, whereas in cancers, elevated WNT2 expression has been detected also in epithelial cells, suggestive of an autocrine signaling loop [67]. Furthermore, elevated WNT2 expression is a common feature in breast carcinomas and breast cancer cell lines [68,69]. It is associated with the epithelial mesenchymal transition, metalloproteinase induction through EGFR transactivation, invasive phenotype, and metastasis [67,69,70]. The oncogene *MYC *is one of the transcriptional targets of the WNT pathway, and it has been shown to enhance its own expression by promoting WNT signaling in tumors [71].

Elevated expression levels of *WNT2, MYC*, its transcriptional co-activator *MAX*, as well as several components of the WNT pathway, suggest that the pathway has a central role in *CHEK2 1100delC-*related tumorigenesis. The genes with elevated expression were involved in canonic and calcium-signaling branches of the WNT pathway. Interestingly, the expression of *LEF1*, the main transcription factor of the pathway, as well as *LRP1*, a cell-surface co-receptor of WNT ligand, was lower in *CHEK2 1100delC *tumors when compared with others. This, together with elevated *E cadherin *(*CDH1*) levels, implies that some balancing forces might act in control of the WNT pathway in the *CHEK2 1100delC *tumors.

*Olfactory receptors *(ORs) form the biggest mammalian gene family, with more than 800 genes present in the human genome and only about 45% of them being functional. ORs are located in clusters that are dispersed across almost all chromosomes. Great interindividual variation exists in the copy number of some OR genes, as well as in the proportion of functional alleles, as a result of low selection pressure [72,73]. ORs bind specifically a wide range of small chemical compounds, including steroid hormones and their analogs, and function in odor recognition [74,75]. Until now, only one OR gene, *prostate-specific G-protein-coupled receptor *(*PSGR/OR51E2*), has been connected to cancer. PSGR is activated by steroid hormones, and the activated receptor has been found to suppress prostate tumor cell proliferation through SAPK/JNK and p38 [75].

In addition to the neurons of the forebrain olfactory bulb [76], ORs are widely expressed across different tissues, but their functions in these locations remain obscure. ORs have been proposed to be involved in cell-cell recognition and in the organization of cells in developing tissues during embryogenesis [77], an analogous function to the one that they have in the organization of the olfactory bulb [78]. But, since OR expression in different cell types does not show a clear pattern that would argue for specific functionality, it has been suggested that *OR *expression would partially result from transcriptional activity of neighboring genes [79]. The existence of neighboring driver genes is one possibility to explain the elevated expression *OR *genes in *CHEK2 1100delC *breast tumors. Our data do not strongly support it, although it cannot be totally excluded. On the basis of our observations, we suggest that the role of the olfactory receptor protein family in cancer cell proliferation and tissue invasion should be investigated further, as these are the two roles in which ORs have been previously implicated.

Converging evidence from the gene-expression and aCGH datasets studied here had the potential to reveal biologic functionalities and pathways that were relevant in *CHEK2 1100delC-*related tumorigenesis. This study concentrated on genomic-level changes that were associated with the *1100delC *germline mutation, whereas previous studies have shed light on the CHEK2 protein expression in breast tumors. The protein expression is reduced or absent in the mutation-carrier tumors, but mechanisms leading to this may vary, and LOH has been reported for only part of *1100delC-*carrier tumors [25,26,32,33]. Here we found that the *CHEK2 *locus was lost in fewer than one third of our mutation-carrier samples and gained in some, with the majority not showing evidence for copy-number variation. We have also determined *CHEK2 *transcript levels in lymphoblastoid cell lines heterozygous for *CHEK2 1100delC *and have shown that constitutive *CHEK2 *gene expression is significantly reduced in cells from heterozygous carriers of *1100delC *(fold change, 0.73; two-sided Student *t*-test *P *= 3.4 × 10^-7^; Muranen et al, unpublished data). These results support the hypothesis of haploinsufficiency being a possible factor driving *CHEK2 1100delC-*related tumorigenesis [80].

We further found an association between the *CHEK2 1100delC *signature of 188 genes and breast cancer relapse. Gene-expression differences of these genes were able to categorize patients into two groups that differed in their risk of breast cancer relapse. Association was observed in all three independent datasets, even though the analysis end points differed slightly between the data from different studies. In the largest dataset, the *CHEK2 1100delC *signature significantly predicted 10-year breast cancer-specific survival. This result is consistent with the previous reports suggesting that the *1100delC *germline genotype associates with worse survival for the carriers [36]. Further studies of larger materials will reveal whether this might be due to a worse therapy response.

Little overlap was found between the *CHEK2 1100delC *gene signature and previously published signatures. For the gene-expression analysis here, a multivariate analysis was used to identify *CHEK2 1100delC-*associated effects that were independent of possible confounding effects, especially the ER status, which is the most important mediator of breast cancer biology and subtypes. The number of genes shared between the published signatures overall is very low, even though they have been shown to perform equally well in predicting clinical outcome. It has been suggested that the prediction capability of these signatures is based on the presence of proliferation-associated genes [81]. Conversely, among the 188 *CHEK2 1100delC-*related genes, only 13 were associated with proliferation. Altogether, this suggests that the *CHEK2 1100delC-*associated signature of 188 genes is distinct from other signatures specifically built to predict breast cancer survival or classify tumors into subtypes and reflects the downstream effects of the germline *CHEK2 1100delC *mutation on tumor progression.

## Conclusions

Altogether, differences between *CHEK2 1100delC *and others tumors were not strong, but we were able to point out chromosomal aberrations and gene-expression changes that were more pronounced in the *CHEK2 1100delC *group. Furthermore, the poor survival associated with the *CHEK2 1100delC *gene-expression signature suggests differential progression pathways in breast tumors with defective *CHEK2*. Our results converge to highlight the importance of the WNT pathway as a driver of tumorigenesis in breast tumors of *CHEK2 1100delC-*mutation carriers. Functional studies are needed to reveal the roles of different components of the pathway in different stages of cancer progression. Another interesting target for future investigation is the role of olfactory receptor family in cancer progression.

## Abbreviations

aCGH: array comparative genomic hybridization; ATM: ataxia telangiectasia mutated; BAC: bacterial artificial chromosome; CDH1: E cadherin; CHEK2: checkpoint kinase 2; CTNND1: catenin delta; DSB: DNA double strand break; ER: estrogen receptor; FFPE: formalin fixed paraffin embedded; GEO: gene expression omnibus; GEX: gene expression; LOH: loss of heterozygosity; MB: megabase; OR: olfactory receptor; PSGR: prostate-specific G protein-coupled receptor.

## Competing interests

The authors declare that they have no competing interests.

## Authors' contributions

HN conceived the study and wrote the manuscript. ÅB conceived the study. RF conceived the study and performed all the steps from nucleic acid extraction to microarray data acquisition. OK and KK performed all the steps from nucleic acid extraction to microarray data acquisition. TM performed the GEX and aCGH data analyses and wrote the manuscript. DG performed the survival analyses, performed the GEX and aCGH data analyses, and wrote the manuscript. PH, CB, and KA contributed samples and clinical information. All authors contributed to and approved the final manuscript for publication.

## Supplementary Material

Additional file 1 **Tumor characteristics of study samples**. Numbers of samples from estrogen-negative and -positive tumors, patients with or without a family history of breast cancer, patients with different rs1800566 genotypes, as well as tumors of different histologic and molecular subtypes in groups of CHEK2 (*checkpoint kinase 2*)-mutation carrier and other tumors for both gene-expression (GEX) and array-comparative genomic hybridization (aCGH) datasets. Molecular subtypes are defined on the basis of Immunohistochemistry results: Luminal A: ER^+^/PR^+^, HER2^-^; Luminal B: ER^+^/PR^+^, HER2^+^; HER2 positive: ER^-^/PR^-^, HER2^+^; Basal: ER^-^/PR^-^, HER2^-^, EGFR^+^; Other triple negative: ER^-^/PR^-^, HER2^-^, EGFR^-^. All data were not available for every sample. EGFR, epidermal growth factor receptor; ER, estrogen receptor; Her2, human epidermal growth factor receptor 2; PR, progesterone receptor. Click here for file

Additional file 2 **Supplementary methods**. [82-94]. Click here for file

Additional file 3 **Unsupervised clustering of array-comparative genomic hybridization (aCGH) and gene-expression (GEX) data**. Left, aCGH samples do not cluster according to any of the covariates. Instead, the clustering is based on informative genomic regions and tumors' overall copy-number profiles. Right, unsupervised hierarchical clustering of gene-expression data suggests that estrogen receptor (ER) status and family history of breast cancer have an impact on the tumor's gene-expression profile. The effect of the other variables is likely to be smaller. Replicates that were used as a quality control cluster together as expected. Positions of *CHEK2 *(*checkpoint kinase 2*), *1100delC-*mutation carrier and control (contr) tumors are indicated by the uppermost color block. *NQO1*^- ^and *NQO1*^+ ^stand for samples with germline *rs1800566 *genotypes: homozygotes of the more common allele (*CC*) and heterozygotes or homozygotes of the rarer allele (*CT *or *TT*), respectively. Frequency of copy-number changes along the genome is depicted with a color scale from blue to red. Blue, few; red, frequent copy-number aberrations. Click here for file

Additional file 4 **Pair-wise correlation plot of aCGH (array-comparative genomic hybridization) quality control (QC) hybridizations**. Heatmap of pair-wise correlation between the hybridization from fresh frozen tissue sample (fresh) and the dye-swap hybridizations from formalin-fixed paraffin-embedded (paraf) tissue. Click here for file

Additional file 5 **Sample-selection criteria and survival-analysis end points in Uppsala, Stockholm, and Rotterdam cohorts**. Click here for file

Additional file 6 **Hierarchical clustering of samples according to 188 differentially expressed genes**. All but two *CHEK2 (checkpoint kinase 2) 1100delC-*mutation carriers cluster together in two branches. Estrogen receptor (ER) status of each sample, either positive or negative, is indicated by the second color block. Click here for file

Additional file 7 **The 862 differentially expressed genes**. Gene identifiers (ID), chromosomal locations, fold change (FC), *P *value, and Benjamini-Hochberg (BH) multiple testing corrected *P *value. Click here for file

Additional file 8 **Functional enrichment of differentially expressed genes with elevated expression levels**. The most frequent annotation categories are involved in olfactory transduction, WNT pathway or zinc finger, Ras-GEF, or EGF protein families. FDR, false discovery rate; Pop, population. Click here for file

Additional file 9 **Differentially expressed genes associated with WNT pathway, cell adhesion, or calcium**. Columns "*CHEK2 *M" and "other M" give average and range of expression values for each gene in *CHEK2 1100delC-*mutation carrier and other tumor samples correspondingly. Click here for file

Additional file 10 **Functional enrichment of differentially expressed genes with reduced expression levels**. The most frequent annotation categories are involved in the protein-expression pathway, mitochondria, or cytoskeleton. (Pop, population; FDR, false discovery rate). Click here for file
