# Supplementary material for: Breast tumors from CHEK2 1100delC-mutation carriers: genomic landscape and clinical implications
Source: Breast Cancer Res. 2011 Sep 20;13(5):R90. doi: 10.1186/bcr3015 (PMC3262202; doi:10.1186/bcr3015)
Supplement: Additional file 1 — Tumor characteristics of study samples. Numbers of samples from estrogen-negative and -positive tumors, patients with or without a family history of breast cancer, patients with different rs1800566 genotypes, as well as tumors of different histologic and molecular subtypes in groups of CHEK2 (checkpoint kinase 2)-mutation carrier and other tumors for both gene-expression (GEX) and array-comparative genomic hybridization (aCGH) datasets. Molecular subtypes are defined on the basis of Immunohistochemistry results: Luminal A: ER+/PR+, HER2-; Luminal B: ER+/PR+, HER2+; HER2 positive: ER-/PR-, HER2+; Basal: ER-/PR-, HER2-, EGFR+; Other triple negative: ER-/PR-, HER2-, EGFR-. All data were not available for every sample. EGFR, epidermal growth factor receptor; ER, estrogen receptor; Her2, human epidermal growth factor receptor 2; PR, progesterone receptor. [file bcr3015-S1.PDF]

Additional file 1. Tumor characteristics of study samples. Numbers of samples from estrogen negative and positive tumors, patients with positive or no family history of breast cancer, patients with different rs1800566 genotypes, as well as tumors of different histological and molecular subtypes in groups of CHEK2 (checkpoint kinase 2) mutation carrier and other tumors for both gene expression (GEX) and array comparative genomic hybridization (aCGH) datasets. Molecular subtypes are defined on the basis of Immunohistochemistry results: Luminal A: ER+/PR+, HER2-; Luminal B: ER+/PR+, HER2+; HER2 positive: ER-/PR-, HER2+; Basal: ER-/PR-, HER2-, EGFR+; Other triple negative: ER-/PR-, HER2-, EGFR-. All data was not available for every sample. (ER: estrogen receptor; PR: progesterone receptor; Her2: human epidermal growth factor receptor 2; EGFR: epidermal growth factor receptor)

	GEX		aCGH	
	CHEK2	other	CHEK2	other
All	13	65	26	76
ER positive	13	48	24	51
ER negative	0	16	2	23
PR positive	11	37	20	42
PR negative	2	27	6	31
HER2 positive	4	6	7	10
HER2 negative	7	41	18	51
EGFR positive	0	9	0	10
EGFR negative	3	21	13	33
Familial	7	34	18	47
Sporadic	5	29	8	27
rs1800566 CC	6	35	18	38
rs1800566 CT/TT	5	21	6	20
Histology:				
Ductal	8	42	23	50
Lobular	3	8	1	13
Medullar	0	2	0	3
Other	0	11	0	8
In situ	2	2	2	2
Molecular subtype:				
Luminal A	7	32	17	36
Luminal B	4	4	6	4
Her2 positive	0	2	1	4
Basal	0	6	0	8
Other triple negative	0	0	0	2
