# Supplementary material for: Breast tumors from CHEK2 1100delC-mutation carriers: genomic landscape and clinical implications
Source: Breast Cancer Res. 2011 Sep 20;13(5):R90. doi: 10.1186/bcr3015 (PMC3262202; doi:10.1186/bcr3015)
Supplement: Additional file 2 — Supplementary methods. [82-94]. [file bcr3015-S2.PDF]

Additional file 2: Supplementary Methods

Tumor tissue samples

Of the 126 breast tumor samples, 84 were fresh frozen tissue specimens stored in -140 °C after surgery whereas 42 were archival formalin fixed paraffin embedded (FFPE) samples. From five patients both fresh and FFPE tissues were available and they were all included as quality controls. Thirteen of the fresh frozen and nineteen of the FFPE samples were taken from altogether thirty tumors of *CHEK2 1100delC* carriers. Tumor characteristics are summarized in Supplementary table 1. In situ tumors were not excluded from the analysis since their profiles did not differ from those of the other tumors. The rare allele of *NQO1* single nucleotide polymorphism (SNP) *rs1800566* has been associated with poor breast cancer survival [46]. The gene is likely to have a role in tumor progression and therefore also the *rs1800566* genotype was included as a covariate in gene expression analysis. The fresh frozen tumor tissue samples were prepared by an experienced breast cancer pathologist to be representative of the tumor tissue. From the FFPE samples an experienced breast cancer pathologist had marked the areas that were highly enriched with tumor tissue.

Nucleic acid extraction

For the gene expression microarray data analysis total RNA was extracted from fresh frozen tissue specimens using a Trizol™ (Invitrogen, CA, USA) based protocol. Briefly, sections of frozen tumor tissues (roughly 10 – 100 mg) were submerged in liquid nitrogen and homogenized using a mechanical homogenizer, after which the tissue powder was dissolved in 4 ml of Trizol™. RNA was then separated into an aqueous phase by addition of 800 ml of chloroform followed by centrifugation at 12,000 g at four degrees centigrade for 15 min. Subsequent RNA extraction and purification was performed using the Qiagen RNeasy RNA purification kit (Qiagen Inc., CA, USA). The quality of the RNA was evaluated by Agilent Bioanalyzer according to the manufacturer’s instructions (Agilent Technologies, CA, USA). RNA was successfully extracted from 78 samples, including thirteen samples from *CHEK2 1100delC* tumors.

For the array comparative genomic hybridization analysis genomic DNA was extracted from both fresh frozen and paraffin-embedded samples. DNA from fresh frozen tissue samples, weighing roughly 10 - 100 mg, was extracted as described previously [40]. 1-4 punches (0.6 mm in diameter) from each paraffin-embedded tissue were combined and

then incubated in xylene to remove the paraffin, followed by an overnight proteinase K treatment. DNA was subsequently extracted using regular phenol-chloroform protocol. Concentration and purity of the extract were assessed by NanoDrop spectrophotometer (NanoDrop Technologies, DE, USA). DNA was successfully extracted from 65 fresh frozen and 42 paraffin embedded samples.

Comparative genomic hybridization microarrays

For the comparative genomic hybridization microarrays, normal human male genomic DNA was used as a reference sample. The samples were labeled using Invitrogens aCGH Bioprime genomic labeling system (Invitrogen, CA, USA), and the labeled products were purified with the CyScribe GFX kit (Amersham Biosciences, Buckinghamshire, UK). Samples from fresh frozen tissues were labeled with Cy3 (Amersham) and hybridized to the arrays together with Cy5-labeled reference. Samples from paraffin-embedded tumor tissues were hybridized twice as dye-swapped duplicates for quality control.

Microarray data acquisition

After hybridization, all slides were scanned using an Agilent Microarray Scanner (Agilent Technologies, CA, USA). Scanned gene expression microarray images were aligned to reporters and manually quality controlled using the GenePix 4.1.1.4 software (Axon Instruments, CA, USA). Spots and regions with excessive background or morphologically aberrant spots were flagged as bad. Arrays with excessive background staining or dissolving of spots, indicative of problems in the hybridization and washing procedure were excluded from further analyses. Subsequent filtering and normalization was carried out in Linear Models for Microarray Data Analysis, *limma* package [43, 82, 83] for R software environment for statistical computing version 2.10 [84]. Background correction was performed as recommended by Ritchie and Silver [45, 85]. Normalization was performed first within arrays with print-tip loess method and thereafter between arrays with the quantile method. Y-chromosomal data was excluded after normalization. Measurements from replicates of the quality controlled probes [18] were merged and used in further analyses.

Scanned CGH microarray images were aligned to reporters and manually quality controlled using the GenePix 4.1.1.4 software as described above. Data quality control was carried out as described previously [40]. Intensity ratios were

normalized on each array with popLowess [86] in BASE data management environment [87, 88] and resulting values manually curated and centered to the mean of the largest population if necessary. After normalization data on X and Y chromosomes were excluded. The data were segmented using circular binary segmentation [89] as implemented in R package *DNAcopy* [90]. Since the aCGH clones were mapped to genome build hg17 and gene expression clones to build hg18, the first mentioned were converted to the more recent build using USCS genome browser's Lift Genome Annotations tool [91]. Clones that did not map to build hg18 were excluded.

Unsupervised clustering of gene expression data

Unsupervised hierarchical clustering was performed according to the Euclidean distances using Ward's linkage for quality control purposes and to detect strong effects related to any covariate. Unsupervised hierarchical clustering did not suggest any major differences between the overall gene expression profiles of *CHEK2 1100delC* mutation carrier and other tumors. However, it revealed that both the tumor's ER status and the patient's positive family history of breast cancer had a marked effect in the gene expression. Almost all ER negative samples clustered within one branch and another branch was occupied by familial samples (Supplementary figure 1). Consequently ER status and family history were taken into consideration in the gene expression multivariate analysis together with other possible confounders, *rs1800566* genotype and tumor's histopathological type. Replicate samples from each of the three tumors, from which there were replicates, clustered closely together implying that gene expression profiles in this experiment were robust and reproducible.

aCGH quality control

From five tumors both paraffin embedded and fresh frozen tissue samples were available. They were used as quality controls to estimate possible technical artifacts derived from different tissue preservation methods. The overall copy number profiles of the parallel hybridizations seemed alike, but there were differences in the proportion of missing values as well as in the magnitude of difference between intensity values of normal and aberrated copy number, possibly as a consequence of different amount of normal-like tissue contamination in the original DNA sample. Similarities of the parallel quality control samples were assessed by estimating the pair wise correlations of called data on the basis of soft calls, i.e. probabilities of gain, loss or normal copy number for each probe [41] (Supplementary figure

2). Overall correlation was calculated as an average of correlations coefficients from pair wise comparisons of probabilities of gain, loss or normal copy number.

If all three parallel hybridizations succeeded (e.g. QC3), the correlation among them was good (Supplementary figure 2). The parallel hybridizations of the less successful samples with greater proportion of missing values or normal tissue contamination had poorer correlation (QC1, QC2 and QC5). QC4 had a normal like copy number profile suggestive of normal tissue contamination and it was removed from further analyses.

From the entire aCGH dataset, samples with normal-like copy number profile were excluded, since they were considered as contaminated by normal tissue. Of the dye-swaps of the paraffin embedded samples only one was selected for further analyses on the basis of the data quality. Samples with higher proportion of probes with successful measurement or more clear-cut copy number populations in the popLowess density plots were included. If the two parallel hybridizations were equally good in these respects, the hybridization where the sample was labeled with cy3 was preferred to minimize technical differences of paraffin samples in comparison to fresh frozen tumor samples that were all labeled with cy3. This resulted in 79 samples, including 22 samples from *CHEK2 1100delC* mutation carriers.

Called tumor aCGH data were clustered according to the soft calls with Weighted clustering of called array CGH data, *wecca* [92], using Ward's linkage and weighing clones according to their variability. *Wecca* is a clustering method especially developed for called aCGH data. It is optimized for clustering ordinal data and it focuses on the informative genomic regions that are not dominated by the normal copy number. *Wecca* clustered the aCGH samples into two major branches (Supplementary figure 1). The most informative aberration for this division was the gain of chromosome 19. In further divisions the aberrations of chromosomes 8, 1 and 5 were among the most important. The most frequent aberrations among all studied breast tumors were 1q gain 8q gain and 16q loss concordantly with previous reports [93, 94]. No obvious clustering pattern for *CHEK2 1100delC* tumors was detected, even though they were slightly enriched in the right branch (Supplementary figure 1): 14 of 36 samples of the right branch were *CHEK2 1100delC* samples, whereas only 8 out of 32 of the left branch. Also tumors with greater number of copy number aberrations were enriched in the right branch. Still the *CHEK2* mutation was not associated to higher frequency of copy number aberrations (Wilcoxon

rank sum test for association p-value 0.45). The clustering was neither associated with any of the other covariates, including ER status, family history of breast cancer, *rs1800566* genotype or histopathological subtype.
