# Supplementary material for: Breast tumors from CHEK2 1100delC-mutation carriers: genomic landscape and clinical implications
Source: Breast Cancer Res. 2011 Sep 20;13(5):R90. doi: 10.1186/bcr3015 (PMC3262202; doi:10.1186/bcr3015)
Supplement: Additional file 3 — Unsupervised clustering of array-comparative genomic hybridization (aCGH) and gene-expression (GEX) data. Left, aCGH samples do not cluster according to any of the covariates. Instead, the clustering is based on informative genomic regions and tumors' overall copy-number profiles. Right, unsupervised hierarchical clustering of gene-expression data suggests that estrogen receptor (ER) status and family history of breast cancer have an impact on the tumor's gene-expression profile. The effect of the other variables is likely to be smaller. Replicates that were used as a quality control cluster together as expected. Positions of CHEK2 (checkpoint kinase 2), 1100delC-mutation carrier and control (contr) tumors are indicated by the uppermost color block. NQO1- and NQO1+ stand for samples with germline rs1800566 genotypes: homozygotes of the more common allele (CC) and heterozygotes or homozygotes of the rarer allele (CT or TT), respectively. Frequency of copy-number changes along the genome is depicted with a color scale from blue to red. Blue, few; red, frequent copy-number aberrations. [file bcr3015-S3.PDF]

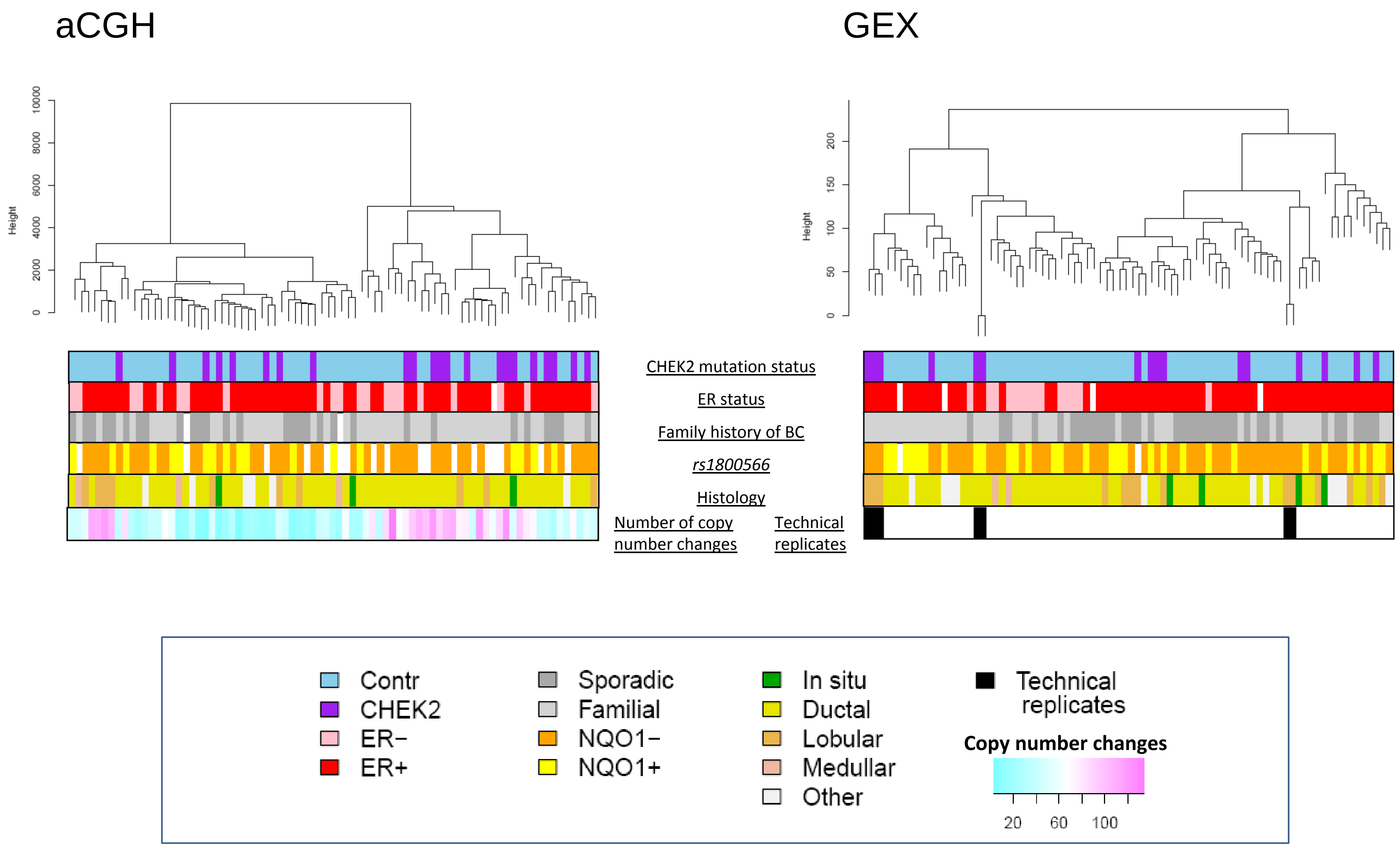

aCGH
GEX
Height
0
2000
4000
6000
8000
10000
0
50
100
150
200
CHEK2 mutation status
ER status
Family history of BC
rs1800566
Histology
Number of copy number changes
Technical replicates
Contr
CHEK2
ER−
ER+
Sporadic
Familial
NQO1−
NQO1+
In situ
Ductal
Lobular
Medullar
Other
Technical replicates
Copy number changes
20
60
100
