# Supplementary figures and images for: Breast tumors from CHEK2 1100delC-mutation carriers: genomic landscape and clinical implications
Source: Breast Cancer Res. 2011 Sep 20;13(5):R90. doi: 10.1186/bcr3015 (PMC3262202; doi:10.1186/bcr3015)

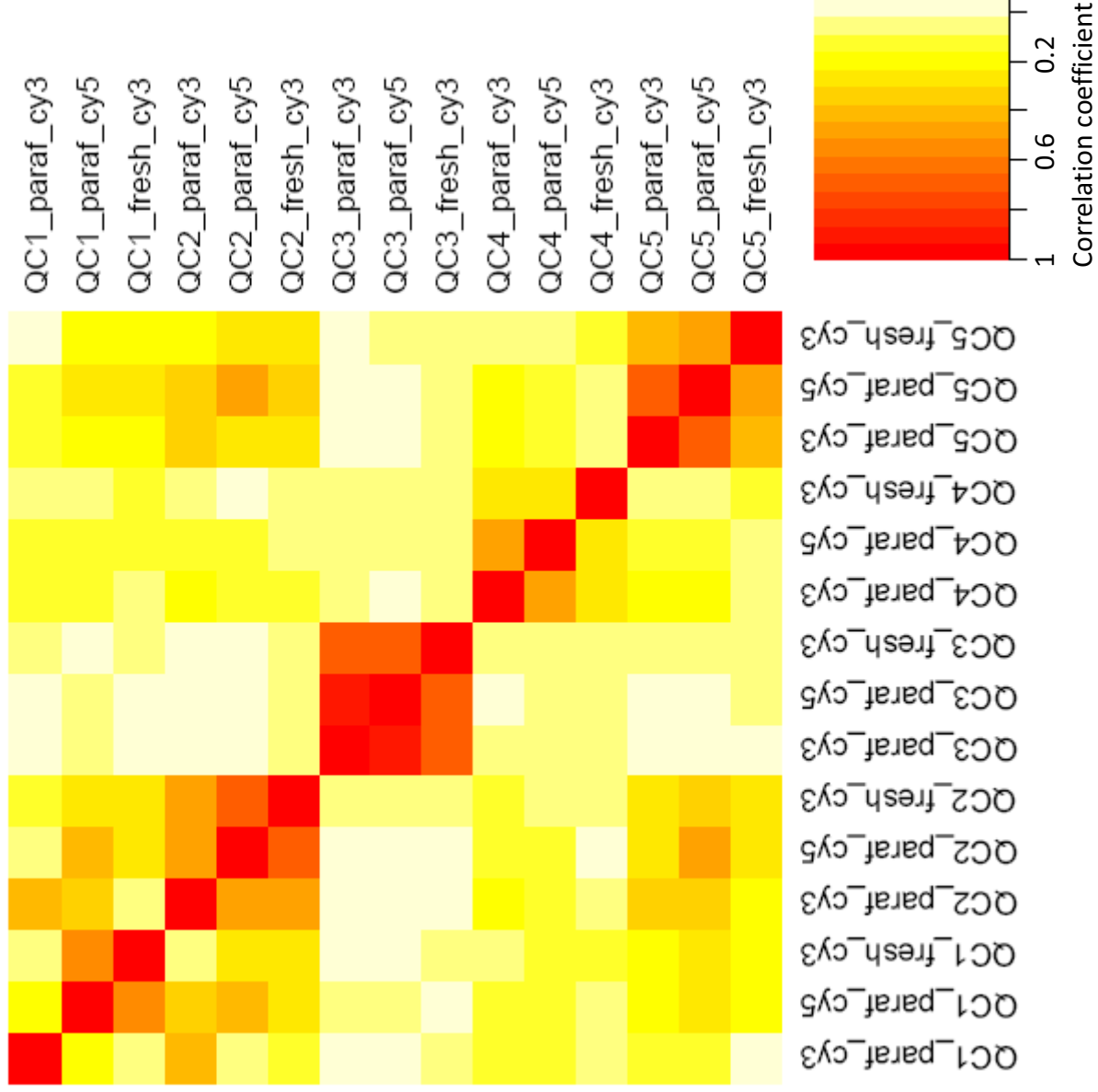

Supplement: Additional file 4 — Pair-wise correlation plot of aCGH (array-comparative genomic hybridization) quality control (QC) hybridizations. Heatmap of pair-wise correlation between the hybridization from fresh frozen tissue sample (fresh) and the dye-swap hybridizations from formalin-fixed paraffin-embedded (paraf) tissue. [file bcr3015-S4.PDF]

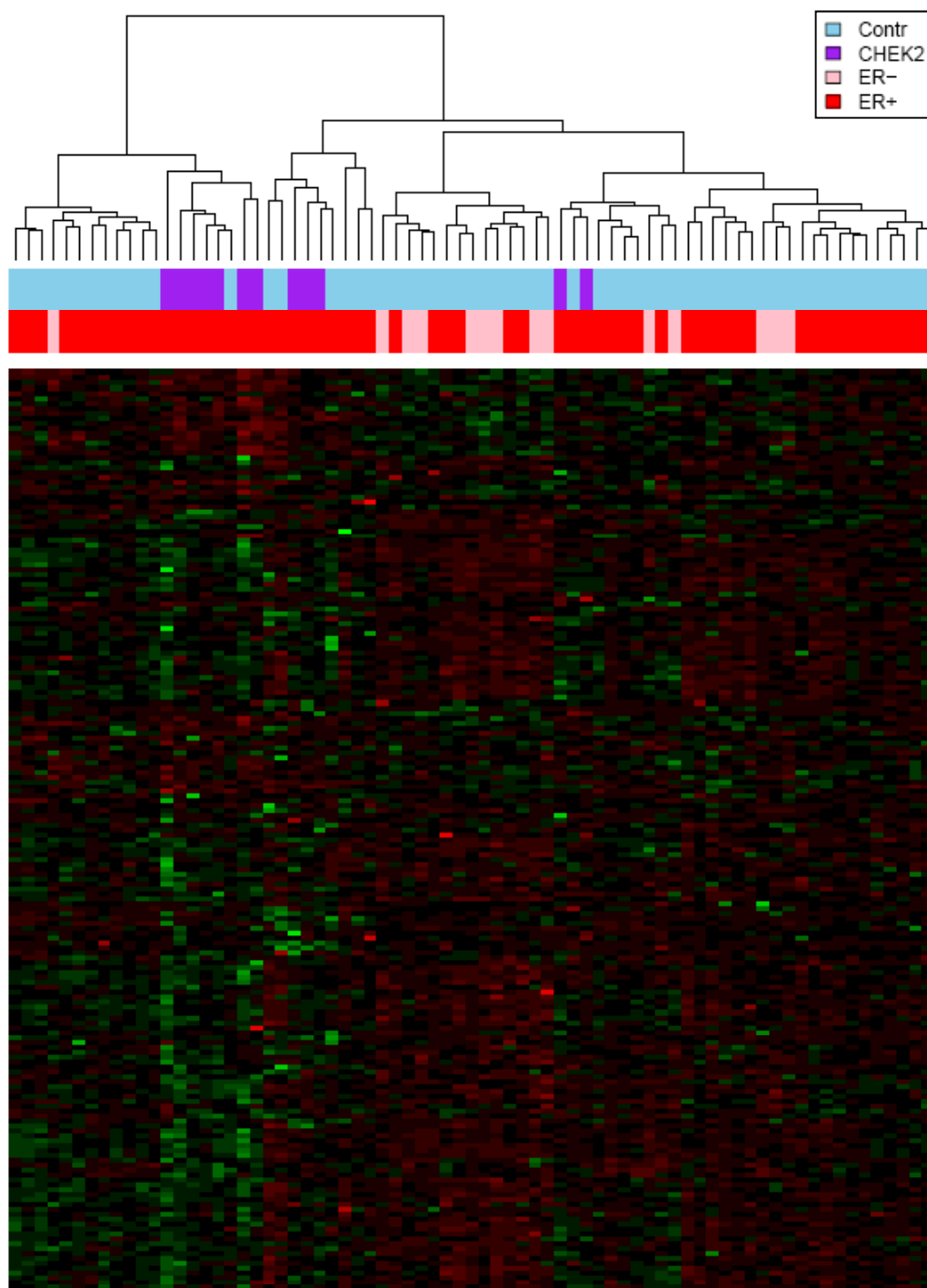

Supplement: Additional file 6 — Hierarchical clustering of samples according to 188 differentially expressed genes. All but two CHEK2 (checkpoint kinase 2) 1100delC-mutation carriers cluster together in two branches. Estrogen receptor (ER) status of each sample, either positive or negative, is indicated by the second color block. [file bcr3015-S6.PDF]
