# Supplementary material for: Breast tumors from CHEK2 1100delC-mutation carriers: genomic landscape and clinical implications
Source: Breast Cancer Res. 2011 Sep 20;13(5):R90. doi: 10.1186/bcr3015 (PMC3262202; doi:10.1186/bcr3015)
Supplement: Additional file 5 — Sample-selection criteria and survival-analysis end points in Uppsala, Stockholm, and Rotterdam cohorts. [file bcr3015-S5.PDF]

Additional file 5. Sample selection criteria and survival analysis end points in Uppsala, Stockholm and Rotterdam cohorts.

	GSE1456	GSE3494	GSE4922	GSE24450
Published	Pawitan et al. 2005	Miller et al. 2005	Ivshina et al. 2006	
Location	Karolinska Hospital, Stockholm	Uppsala county	Uppsala county	Helsinki University Central Hospital
Number of samples	159	251	249	183
Collection periods	1994-1996	1987-1989	1987-1989	1997-1998, 2000, 2001-2004
Selection criteria	Unselected primary tumors, non-invasive tumors excluded	Unselected primary tumors	Unselected primary tumors	151 breast tumors from three unselected cohorts, 32 breast tumors from familial cases
Survival analysis events	Breast cancer related death;<br>Breast cancer relapse	Breast cancer related death	Breast cancer related death or any relapse (local, regional or distant)	Breast cancer death within 10 years;<br>Breast cancer death or distant metastasis in 5 years
Array	Affymetrix Human Genome U133A or U133B Array	Affymetrix Human Genome U133A or U133B Array	Affymetrix Human Genome U133A or U133B Array	Illumina HumanHT-12 v3
Proportion of the 188 CHEK2 related genes on the array	108 (57.4%)	108 (57.4%)	108 (57.4%)	131 (69.7%)
